# Cooperative control between AtRGS1 and AtHXK1 in a WD40-repeat protein pathway in *Arabidopsis thaliana*

**DOI:** 10.3389/fpls.2015.00851

**Published:** 2015-10-13

**Authors:** Jian-Ping Huang, Meral Tunc-Ozdemir, Ying Chang, Alan M. Jones

**Affiliations:** ^1^College of Life Science, Northeast Agricultural UniversityHarbin, China; ^2^Department of Biology, University of North Carolina, Chapel HillNC, USA; ^3^Department of Pharmacology, University of North Carolina, Chapel HillNC, USA

**Keywords:** plant heterotrimeric G protein, sugar signaling, *Arabidopsis* regulator of G-protein signaling 1 protein (AtRGS1), hexokinase 1 (HXK1)

## Abstract

HEXOKINASE 1 (AtHXK1) and Regulator of G-protein Signaling 1 (AtRGS1) pathways, mediate D-glucose signaling in *Arabidopsis*. However, it is not known the degree, if any, that these pathways overlap and how. We show modest signaling crosstalk between these pathways, albeit complex with both epistatic interactions and additive effects that may be indirect. The action of HXK1 on AtRGS1 signaling lies downstream of the primary step in G protein-mediated sugar signaling in which the WD-repeat protein, AGB1, is the propelling signaling element. RHIP1, a previously unknown protein predicted here to have a 3-stranded helical structure, interacts with both AtRGS1 and AtHXK1 *in planta* and is required for some glucose-regulated gene expression, providing a physical connection between these two proteins in sugar signaling. The *rhip1* null mutant displays similar seedling growth phenotypes as *rgs1-*2 in response to glucose, further suggesting a role for RHIP1 in glucose signaling. In conclusion, glucose signaling is a complex hierarchical relationship which is specific to the target gene and sugar phenotype and suggests that there are two glycolysis-independent glucose signaling sensors: AtRGS1 and AtHXK1 that weakly communicate with each other via feed-back and feed-forward loops to fine tune the response to glucose.

## Introduction

Glucose affects plant growth and development by acting as both a metabolite and a signaling molecule (Sheen, 2014). In *Arabidopsis thaliana*, glucose is sensed by (1) a plasma membrane G-protein-coupled pathway involving *REGULATOR OF G-PROTEIN SIGNALING 1* (AtRGS1), (2) a *HEXOKINASE 1* (AtHXK1) pathway, and (3) a glycolysis-dependent *SNF1-RELATED KINASE 1*/*TARGET OF RAPAMYCIN* (SnRK1/TOR) pathway. On the *Arabidopsis* plasma membrane, the G protein complex contains the core G-protein subunits Gα (AtGPA1), Gβ (AGB1) and Gγ (AGG1), three WNK kinases (AtWNK1, AtWNK8, and AtWNK10) and the 7-transmembrane AtRGS1 protein. AtRGS1 keeps the core G protein complex in its inactive (GDP-bound) state and physical decoupling of AtRGS1 from AtGPA1 allows this G protein to self-activate by spontaneously binding GTP. Sustained activation of glucose signaling depends, in part, on removal of the inhibiting AtRGS1 protein by endocytosis of this plasma membrane-anchored glucose sensor (Urano et al., 2012; Fu et al., 2014). The WD40 repeat subunit of the G protein AGB1 recruits the WNK kinases which phosphorylate AtRGS1 consequently triggering the endocytosis event.

AtRGS1-dependent G-protein-coupled signaling regulates many cellular biological processes, such as cell growth and proliferation (Chen et al., 2006a; Booker et al., 2010; Urano et al., 2012), abiotic stress tolerance (Chen et al., 2006b; Colaneri et al., 2014), stomatal density (Zhang et al., 2008), chloroplast development (Zhang et al., 2009), seed germination (Chen et al., 2006c), morphogenesis and development (Chen and Jones, 2004; Chen et al., 2006b). Specifically, loss of *AtRGS1* confers increased growth and high glucose tolerance in *Arabidopsis*. Loss-of-function mutations in the WD40-repeat subunit AGB1 have the most severe phenotypes.

AtHXK1 regulates glucose-responsive gene transcription directly by forming a nuclear co-repressor complex with the vacuolar H+-ATPase B1 (VHA-B1) and the 19S regulatory particle of the proteasome subunit (RPT5B) (Cho et al., 2006). The *hxk1* (aka *gin2*-1), *vha-b1* and *rpt5b* null mutants display similar seedling and adult plant phenotypes (e.g., reduced growth in roots, leaves and inflorescences), and glucose response defects (namely, insensitivity to high glucose-mediated developmental arrest) (Moore et al., 2003; Cho et al., 2006).

The glycolysis-dependent SnRK1/TOR pathway contains two protein kinases SnRK1 and TOR that may function as cellular energy sensors instead of sugar sensors (Lastdrager et al., 2014; Tome et al., 2014). TOR is activated and promotes growth in response to favorable nutritional and energy conditions, while SnRK1 is stimulated upon nutrient and energy starvation conditions (Lastdrager et al., 2014; Tome et al., 2014).

Until now, the mechanisms within each of these glucose signal transduction pathways were studied independently of each other, yet, analogous to the integrative HXK/G protein mechanism for glucose sensing in yeast (Rolland et al., 2000; Kim et al., 2013), it is likely that an integration of these pathways is essential for plants to make a fast and coordinated response to extracellular and intracellular glucose conditions. In this study, we focused on functional crosstalk between AtRGS1- and AtHXK1-dependent sugar signaling (Moore et al., 2003; Chen and Jones, 2004; Urano et al., 2012). By comparing the expression of a selected set of glucose-regulated genes as well as a series of physiological sugar-related phenotypes, we determined that the two glucose sensors AtRGS1 and AtHXK1 communicate via cooperative loops in determining the final response to glucose. This occurs downstream of AtRGS1 endocytosis. RGS1-HXK1 INTERACTING PROTEIN 1 (RHIP1) serves as the physical scaffold for these two sensors.

## Materials and Methods

### Plant Materials

The T-DNA insertion mutant *rgs1*-2 was described by Chen et al. (2003), and the ethylmethane sulfonate (EMS)-mutagenized mutant *gin2*-1 was described by Moore et al. (2003). The *35S::RGS1-YFP* over-expression line was previously described by Urano et al. (2012). *hxk1*-1 (*SALK_034233*), *hxk1*-2 (*CS864200*), *hxk1*-3 (*CS861759*), *rhip1*-1 (*SALK_091518*), and *rhip1*-2 (*SALK_061002*) mutants were obtained from the *Arabidopsis* Biological Resource Center (ABRC) stock. Plants homozygous for *hxk1*-1, *hxk1*-2, *hxk1*-3, *rhip1*-1, and *rhip1*-2 were isolated by PCR using genomic DNA with T-DNA insertion verification primers and the insertion was confirmed by sequencing. Reverse transcriptase polymerase chain reaction (PCR) was used to analyze the *AtHXK1* or *RHIP1* transcripts in these five mutants. The primers used are shown in Supplementary Table S1. Our analyses disproved an earlier claim by Reda (2013) that *hxk1*-1 (*SALK_034233C*) lacked expression of *HXK1* (Supplementary Figure S1); *hxk1*-1 is not a null mutant as previously reported and the reported phenotypes of the *hxk1*-1 mutant should be re-interpreted. The open reading frame of *AtRGS1* in pEarleyGate101 vector (C-terminal *YFP-HA*) was previously described by Urano et al. (2012). The *35S::AtRGS1-YFP* over-expression lines in the *hxk1*-3, *rhip1*-1 or *rhip1*-2 mutant background were generated by *Agrobacterium*-mediated transformation (Bechtold et al., 1993), and T2 generation seed was used. The *pCDKA;1::GUS* reporter was crossed to *rgs1*-2 and *hxk1*-3 single mutant plants, and F3 and F4 generation plants were used for phenotype analysis. All mutants used are in the ecotype Columbia (Col-0) except *gin2*-1 which is in the Landsberg (Ler) background.

### Quantitative Real-time PCR (qRT-PCR)

Quantitative real-time PCR was performed exactly as described by Grigston et al. (2008) and Urano et al. (2012). Briefly, ∼150 seeds were cultured in 75 ml 1/2 × MS liquid media with 1% sucrose at 23°C shaking (∼140 rpm) under constant low light (70 μmol s^-1^m^-2^) conditions. After 7 days, the seedlings were washed in sterilized water three times, and then starved in 1/2 × MS liquid media lacking sugar for 2 days in the dark. The seedlings were treated with fresh 1/2 × MS liquid media containing 0% (control), or 3% (w/v) D-glucose for 3 h in dark conditions, then harvested by flash freezing in liquid N_2_. The mRNA and cDNA were prepared with RNAeasy^TM^ (Qiagen) and Superscript III (Invitrogen), respectively, according to the manufacturer’s instructions. Expression of *TBL26, HXK1, RGS1, CA2, CAB2, DIN1*, and *TUB4* was analyzed by qRT-PCR with SYBRgreen (Invitrogen). The comparative CT (*T*hreshold *C*ycle defined as the cycle number at which the fluorescence generated within a reaction crosses the threshold line) method described in detail in Livak and Schmittgen (2001), was performed for qRT-PCR. First, validation experiments demonstrated that the targets (*TBL26, HXK1, RGS1, CA*2, *CAB*2, *DIN1*) and endogenous control (*TUB4*) have single amplification products determined by gel electrophoresis and relatively equivalent PCR efficiencies (relative standard curve). Second, samples from each biological repeat were run in triplicate on a single plate. The CT mean and standard deviation values of the three replicate sample results were calculated. The ΔCT value was calculated by:

$$\Delta {CT=CT}_{\mathrm{target}\;\mathrm{gene}}\;-\;{\mathrm{CT}}_{\mathrm{endogenous}\;\mathrm{control}\;\mathrm{gene}}$$

The standard deviation of the ΔCT was calculated by:

$$S\;=\;{\left(S^{2}{\;}_{\mathrm{target}\;\mathrm{gene}}\;+\;S^{2}{}_{\mathrm{endogenous}\;\mathrm{control}\;\mathrm{gene}}\right)}^{1/2};\;(S\;=\;\mathrm{standard}\;\mathrm{deviation})$$

The ΔΔCT was calculated by:

$$\Delta \Delta \mathrm{CT}\;=\;\Delta \mathrm{CT}{\;}_{\mathrm{test}\;\mathrm{sample}}\;-\;\Delta \mathrm{CT}{\;}_{\mathrm{control}\;\mathrm{sample}}$$

The standard deviation of the ΔΔCT value is the same as the standard deviation of the ΔCT value. Fold-differences between test sample and control sample was calculated by 2^-ΔΔCt^ with ΔΔCT + S and ΔΔCT - S, where S is the standard deviation of the ΔΔCT value. Fold-differences calculated using ΔΔCT method are shown as a range from 2^-ΔΔCt+s^ to 2^-ΔΔCt-s^.

### Plant Growth Assays

Seeds were sown onto soil and stratified at 4°C for 3 days, and then transferred to a 23°C growth chamber in 16/8 h (L/D). Two or 3-week-old seedlings were photographed. Alternatively, seeds were surface-sterilized with 70% ethanol and 95% ethanol for 10 min each, then sown onto 1/4 × MS with 0.5% phytoagar (pH adjusted to 5.75 with 5 N KOH). Seeds were stratified at 4°C in the dark for 3 days, germinated and grown horizontally at 23°C with a 16/8 h (L/D) cycle (70 μmol s^-1^m^-2^). Three days later, the seedlings were transferred to 1/4 × MS with 0.5% phytoagar (pH adjusted to 5.75 with 5 N KOH) containing 0, 0.25, 0.5, 0.75, 1, 2, or 3% (w/v) D-glucose, and grown vertically for root growth analysis. Every 24 h, the root tips were marked. The root lengths were calculated using ImageJ software. The “green seedling” assay was performed as described by Moore and coworkers (Moore et al., 2003). Briefly, surface-sterilized seeds of Col-0, *rgs1*-2, *hxk1*-3, *hxk1-*3/*rgs1-*2, and *rhip1-*2 mutant were sown onto 1/4 × MS with 0.5% phytoagar containing 6% (w/v) D-glucose or D-mannitol, stratified at 4°C in the dark for 3 days, germinated and grown horizontally at 23°C with continuous light (70 μmol s^-1^m^-2^) for 10 days. The average percentage of seedlings showing green cotyledons was determined. The “green seedling” assay in response to saline stress was performed as described by Colaneri et al. (2014). Briefly, surface-sterilized seeds were germinated and seedlings grown on 1/4 MS agar media supplemented with 125 mM NaCl and 0.5% D-glucose (w/v). Green seedlings per plate (36 seeds per plate) were counted 10 days after germination.

### β-Glucuronidase (GUS) Staining Assay

*Arabidopsis* seeds were surface-sterilized and stratified as described above. Seeds germinated in 24-well plates containing 1 ml 1/2 MS liquid medium (pH adjusted to 5.75 with 5 N KOH) on a shaker (∼30 rpm) at 23°C under continuous low light (70 μmol s^-1^m^-2^). 3-day-old seedlings were then transferred to 1 ml fresh 1/2 MS liquid medium supplemented with 3% (w/v) D-glucose or D-mannitol (pH adjusted to 5.75 with 5 N KOH) for 3 more days in culture.

GUS staining was performed following the method described by Malamy and Benfey (1997). Seedlings were infiltrated in 100 mM Tris-HCl (pH 7.5) buffer containing 2.9 mg/ml NaCl, 20% (v/v) methanol, 0.001% (v/v) Triton X-100 and 0.5 mg/ml X-gluc (5-Bromo-4-chloro-3-indoxyl-beta-D-glucuronide cyclohexyl ammonium salt; Gold Biotechnology, Inc.). After incubation for 12–18 h at 37°C in dark, seedlings were cleared in 70% ethanol and photographed on a Nikon inverted microscope DIAPHOT-TMD. ImageJ software was used for analysis.

### Informatic Methods

The protein sequence of RHIP1 (At4g26410) was obtained by searching against the *Arabidopsis thaliana* protein database supported by The *Arabidopsis* Information Resource (TAIR). The RHIP1 protein sequence was submitted to I-TASSER (Iterative Threading ASSEmbly Refinement) online^1^, to generate the predicted RHIP1 protein structure and the GO biological process of RHIP1 protein based on global and local protein similarity. PyMOL(TM) Molecular Graphics System (Version 1.7.0.0.) was used to generate the final high-quality protein structure model image based on the data of model 1 from I-TASSER. GO annotations of proteins directly interacting with RHIP1 were retrieved from TAIR GO Annotation Search^2^, and functional categorization by their TAIR loci for GO molecular function or GO biological process were expressed as percent of total.

### AtRGS1-YFP Internalization Analysis

Fluorescence quantification for AtRGS1-YFP internalization was performed as described by Urano et al. (2012) and Fu et al. (2014). Sterilized, stratified seeds were germinated in 6-well plates containing 2 ml 1/2 × MS liquid medium (pH adjusted to 5.75 with 5 N KOH) at 23°C under darkness. Seedlings (7-day-old) were treated with 0 or 3% D-glucose (w/v) for 30 min. Hypocotyl epidermal cells located 2–4 mm below the cotyledon were imaged (Z stacks obtained) using a Zeiss LSM710 confocal laser scanning microscope equipped with a 20× Plan-NeoFluor (N.A. = 0.5) objective and a 40× C-Apochromat (N.A. = 1.20) water immersion objective. YFP fluorescence was excited by a 514 nm argon laser and detected at 526–569 nm by a photomultiplier detector. At least 10 sets of images from 5 seedlings were obtained for internalization quantification analysis by ImageJ software.

### Bimolecular Fluorescence Complementation (BiFC)

Bimolecular fluorescence complementation was performed as described in Klopﬄeisch et al. (2011). The entire open reading frames of *RHIP1* or *AtHXK1* were amplified by PCR from a cDNA library made from seedlings in qRT-PCR analysis. Open reading frames were subcloned into the p-ENTR/D-TOPO vector (Invitrogen, Carlsbad, CA, USA) and recombined into the BiFC vectors pBatTL-sYFP-N and pBatTL-sYFP-C (C-terminal split-nYFP and cYFP tag, respectively), and pCL112 and pCL113 (N-terminal split-nYFP and cYFP tag, respectively) by LR recombination reaction. The open reading frame of *AtRGS1* in BiFC vectors was previously described by Grigston et al. (2008). Split nYFP- and cYFP-tagged protein pairs, p19 (gene silencing suppressor), and Mt-rk (mitochondrial RFP marker, an internal transformation control) were co-expressed in 4- to 5-week-old *Nicotiana benthamiana* leaves by *Agrobacterium tumifaciens*-mediated infection. Tobacco leaf epidermal cells were imaged using a Zeiss LSM710 confocal laser scanning microscope equipped with an Apochromat 40 × water-emersion objective (N.A. = 1.2). YFP fluorescence was excited by a 514 nm argon laser and detected at 526–569 nm by a photomultiplier detector, and RFP fluorescence was excited by a diode laser and detected at 565–621 nm.

## Results and Discussion

### AtRGS1 and AtHXK1 Co-regulate the Expression of a Group of Glucose-regulated Genes

**FIGURE 1:**
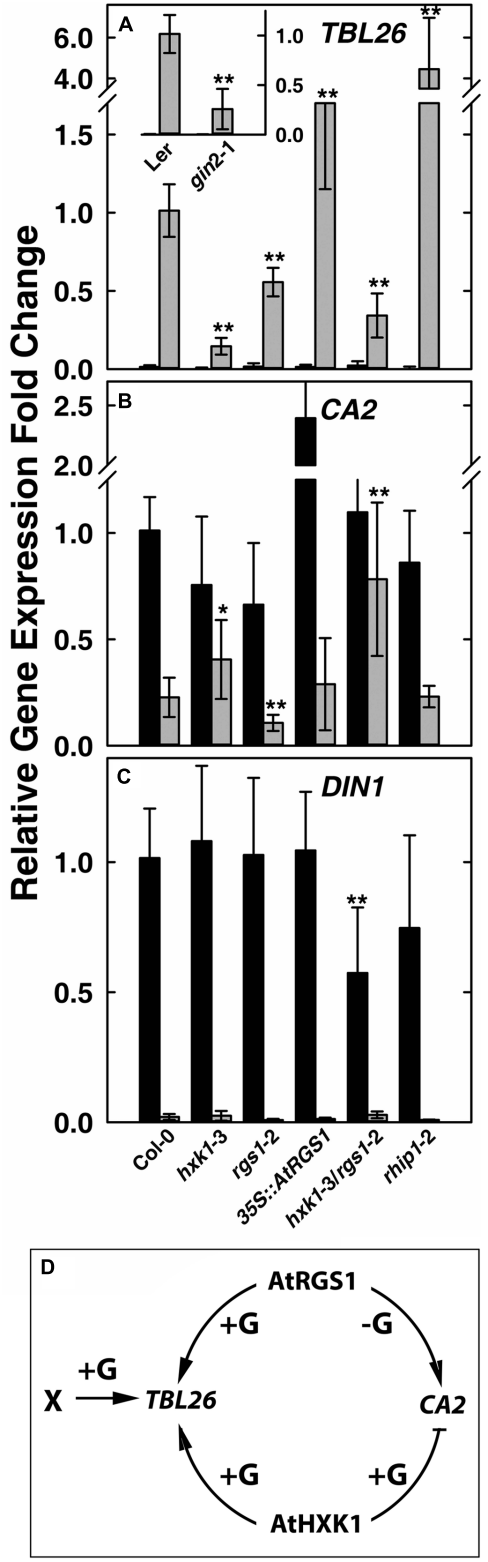
**Quantitative Real-time PCR (qPCR) analysis of glucose-reporter genes *TBL26, CA2* and *DIN1* in *Arabidopsis* seedlings and the corresponding mechanism models.** Seven-day-old seedlings with the indicated genotypes indicated at the bottom of panels **(A–C)** were starved for 2 days then treated with 1/2 × MS medium (0% D-glucose, shown by black bars) or 3%D-glucose (gray bars) for 3 h as described in Section “Materials and Methods.” Transcripts of *TBL26* **(A)**, *CA2* **(B)**, and *DIN1* **(C)** were quantitated using qRT-PCR. Values are the means of the fold changes ± SD of 3–5 independent biological replicates. Each biological replication had at least three technical replications. ANOVA single factor analysis (α = 0.05) was conducted to compare the relative fold-change in gene expression of different plant lines with wild type control plants. ^∗^*P* < 0.05; ^∗∗^*P* < 0.01. **(D)** Simplest model accounting for the gene expression data based on the mutant behaviors. +G, addition of D-glucose; -G, no glucose.

Glucose induces in an AtRGS1-dependent manner the expression of *TRICHOMELESS 26* (*TBL26*, At4g01080), *JACALIN-RELATED LECTIN 7* (*JAL7*, At1g52040), *JACALIN-RELATED LECTIN23* (*JAL23*, At2g39330), At1g54020 (*JAL* gene) among ∼30 genes (Grigston et al., 2008; Urano et al., 2012). The time course and dose responsiveness for regulation of *TBL26* is the best documented among the small set of genes regulated by AtRGS1. Even fewer genes to date are documented to be regulated by HXK1 (Sheen et al., 1999). The best characterized is the involvement of AtHXK1 in glucose repression of the *CARBONIC ANHYDRASE 2* (*CA2*, At5g14740) and the photosynthetic gene *CHLOROPHYL A/B-BINDING 2* (*CAB2*, At1g29920) (Moore et al., 2003; Cho et al., 2006). As shown in **Figure 1A** inset, the D-glucose induced expression of *TBL26* was severely depressed in the *hxk1* mutant (*gin2*-1 in the Ler background) with ∼80% difference between *TBL26* expression in *Ler* and in *gin2*-1, suggesting an active role of AtHXK1 in glucose-induced *TBL26* expression. All other studies on *TBL26* were conducted using the Col ecotype. In order to exclude the influence of different ecotypes, it was necessary to generate a transcript-null allele (*hxk1*-3) in the *Columbia* ecotype (Supplementary Figure S1). Glucose-induced expression of *TBL26* was impaired in seedlings lacking either *AtRGS1* or *AtHXK1* (**Figure 1A**), and this impairment was not the result of lowered expression of *AtRGS1* in the *hxk1*-3 mutant, or of *AtHXK1* in the *rgs1*-2 mutant (Supplementary Figure S2), suggesting an overlapping or additive function between AtRGS1 and AtHXK1 in glucose-regulated gene expression. The level of *TBL26* expression in the *hxk1*-3 mutant was lower than in the *rgs1*-2 mutant (*P* < 0.01) but combining these alleles conferred statistically similar level as the *rgs1*-2 mutant indicating that the *rgs1*-2 allele is epistatic to the *hxk1*-3 allele. Note, however, that loss of both AtRGS1 and AtHXK1 did not completely eliminate glucose induction of *TBL26* expression indicating that a third (glucose) input must exist. AtRGS1 slightly inhibits *TBL26* expression but also positively reinforced AtHXK1 although with a different weight than inhibition.

Glucose represses some gene expression in an AtHXK1-dependent manner (Sheen, 1990; Koch, 1996). We next examined whether AtRGS1 influences AtHXK1-mediated gene repression of *CA2* gene expression. Glucose-repression of *CA2* was slightly attenuated in the *hxk1* mutants (both Col and Ler alleles), consistent with previous studies (Moore et al., 2003; Cho et al., 2006). The *rgs1*-2 mutant displayed slightly lower *CA2* gene expression with (*P* = 0.003236) or without (*P* = 0.00223) glucose treatment (**Figure 1B**), suggesting that AtRGS1 promotes *CA2* basal gene expression. To test this hypothesis, we examined the effect of over-expression of AtRGS1 on the basal expression of *CA2*. The basal *CA2* expression without glucose treatment was strongly increased to nearly 2.5-fold in the *35S::AtRGS1* plants (*P* = 1.25E-05), while the glucose-repressed level of expression was similar to wild type. The *hxk1-*3/*rgs1-*2 double mutant had statistically the same basal level of *CA2* expression (*P* > 0.05) but the glucose repression was attenuated although statistically to no more or less degree than the in the *hxk1*-3 mutant (*P* < 0.025) (**Figure 1B**). The epistasis analysis did not provide strong evidence for a heirarchial role of AtRGS1 in AtHXK1 regulation of *CA2* expression, however, the large effect of over-expression of AtRGS1 is suggestive of cooperation between these two pathways although with the accompanying caveats associated with interpreting gain-of-function alleles. We also examined another AtHXK1-mediated gene repression, namely *CAB2*, and obtained a similar result as for *CA2* (Supplementary Figure S3). AtHXK1 mediates the main pathway toward *CA2* repression. AtRGS1, based on the gain-of-function data, induces *CA2* gene expression in the absence of glucose. An inhibitory genetic interaction by AtHXK1 on AtRGS1 fits the observed result. There is no need to implicate an independent glucose pathway since loss of both AtRGS1 and AtHXK1 statistically eliminated glucose repression of *CA2* expression.

As discussed above, there are at least three sugar-sensing pathways in *Arabidopsis*, the two examined above and the third being the SnRK1/TOR pathway tested next. Glucose-induced repression of *DARK INDUCIBLE* 1 (*DIN1*, At4g35770) is regulated by glycolysis-dependent SnRK1(Baena-Gonzalez et al., 2007; Baena-Gonzalez and Sheen, 2008). As shown in **Figure 1C**, there was no obvious difference in glucose repression of *DIN1* among Col-0, *hxk1*-3, *rgs1*-2, and *35S::AtRGS1* over-expression lines, suggesting no role for either HXK1 or AtRGS1 in glucose-regulated *DIN1* expression. However, the *hxk1*-3*/rgs1*-2 double mutants had a lower basal level of *DIN1* expression (*P* = 0.007405), implying redundancy or, more likely, an indirect effect on the glucose economy.

The behavior of the *hxk1, rgs1*, and *hxk1*/*rgs1* mutants in glucose-regulated gene expression paints a complex relationship between two glucose sensing pathways in *Arabidopsis* and clearly indicate that these two pathways do not operate independently without at least modest influence on each other through direct or indirect mechanisms. The epistasis analyses suggest that AtHXK1 and AtRGS1 operate cooperatively for *TBL26* and *CA2* regulation (**Figure 1D**) and redundantly or not at all in *DIN1* regulation. This unusual genetic relationship is born out in the developmental phenotypes of the mutants described below.

### AtRGS1 Genetically Interacts with AtHXK1 in Modulating Early Seedling Development

**FIGURE 2:**
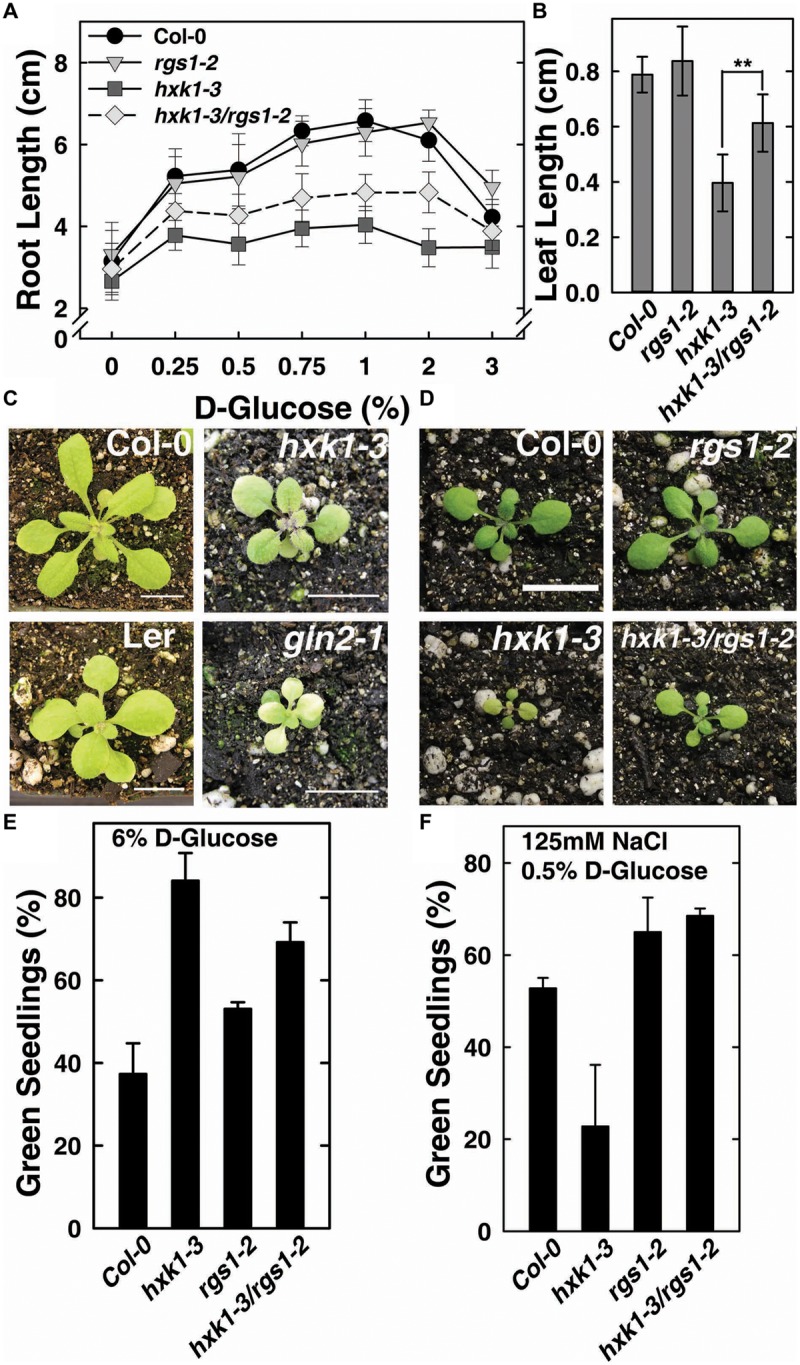
***hxk1*-3*/rgs1*-2 double mutants show intermediate growth phenotype between *hxk1*-3 and *rgs1*-2 sngle mutants. (A)** Comparison of root elongation of Col-0, *rgs1*-2, *hxk1*-3, and *hxk1*-3/*rgs1*-2 mutant seedlings transferred to1/4 MS medium supplemented with 0, 0.25, 0.5, 0.75, 1, 2 or 3% D-glucose (w/v) under low intensity light (70 μmol s^-1^ m^-2^) in long-day chamber in 16/8 h (L/D). Values are means ± SD (*n* = 10–18). **(B)** Comparison of leaf size (the third leaf) of 2-week-old Col-0, *rgs1*-2, *hxk1*-3, and *hxk1*-3/*rgs1*-2 mutant seedlings grown under light (160 μmol s^-1^ m^-2^) in 16/8 h (L/D). Values are means ± SD (*n* = 10–12). ANOVA single factor analysis (α = 0.05) was conducted to compare the leaf size in *hxk1*-3and *hxk1*-3/*rgs1*-2. ^∗∗^*P* < 0.01. **(C)** 3-week-old Col-0, *hxk1*-3, Ler, and *gin2*-1 plants grown under high intensity light (160 μmol s^-1^ m^-2^) in 16/8 h (L/D). Scale bar = 1 cm. **(D)** 2-week-old Col-0, *hxk1*-3, *rgs1*-2, and *hxk1*-3/*rgs1*-2 mutant plants grown under high intensity light (160 μmol s^-1^ m^-2^) in 16/8 h (L/D). Scale bar = 1 cm. **(E)** Green seedling assay of the Col-0, *rgs1*-2, *hxk1*-3, and *hxk1*-3/*rgs1*-2 mutant. The assay was performed as described in Section “Materials and Methods.” The average percentage of seedlings showing green cotyledons was determined and presented with means ± SD from one representative experiment of 4 biological replications. **(F)** Col, *rgs1*-2, *hxk1-3*, and *rgs1-2/hxk1-3* have altered responses to saline stress. The assay was performed as described in Section “Material and Methods.” Values are means ± SD from quintuplicate.

The feed-back regulation of AtHXK1 and AtRGS1 on each other in glucose-regulated gene expression predicts that loss of either would confer opposite physiological phenotypes while loss of both would confer a phenotype shared by neither (e.g., an intermediate phenotype). Previous reports indicate that genetic ablation of *AtHXK1* reduces growth of roots, leaves and inflorescences in the Ler ecotype (Moore et al., 2003; Cho et al., 2006), whereas, *rgs1*-2 null mutants have longer roots, hypocotyls, and greater rosette diameter than wild type (Chen et al., 2003; Urano et al., 2013). In order to study the different roles between AtHXK1 and AtRGS1 in glucose-regulated primary root growth, 3-day-old *Arabidopsis* seedlings germinated in 1/4 MS solid medium containing 0% glucose were transferred to different glucose conditions, and the lengths of primary roots on the 10th day was measured. As shown in **Figure 2A**, compared to wild type plants, *hxk1*-3 mutants had shorter roots under all glucose conditions; *rgs1*-2 mutants had similar root lengths to Col-0; *hxk1*-3*/rgs1*-2 double mutants had intermediate root elongation between the *rgs1*-2 and *hxk1*-3 mutants. The *rgs1*-2 allele rescued the *hxk1*-3 allele in leaf and rosette size (**Figures 2B–D**). Given that root elongation and leaf extension are all based on cell division in the meristem and subsequent cell expansion, the intermediate phenotype of *hxk1*-3*/rgs1*-2 double mutants imply that the two glucose sensors—AtRGS1 and AtHXK1 have opposite roles in regulation of cell proliferation. Glucose and salt stress responsiveness were also measured (**Figures 2E,F**). Both *hxk1*-3 and *rgs1*-2 were hyposensitive to a high dose of glucose but this hyposensitivity was not additive. The *hxk1*-3 allele conferred hypersensitivity to NaCl while the *rgs1*-2 allele and the double mutant were less sensitive. The *rgs1*-2 allele is epistatic to the *hxk1*-3 allele indicating that AtRGS1 and AtHXK1 operate in the same genetic pathway.

**FIGURE 3:**
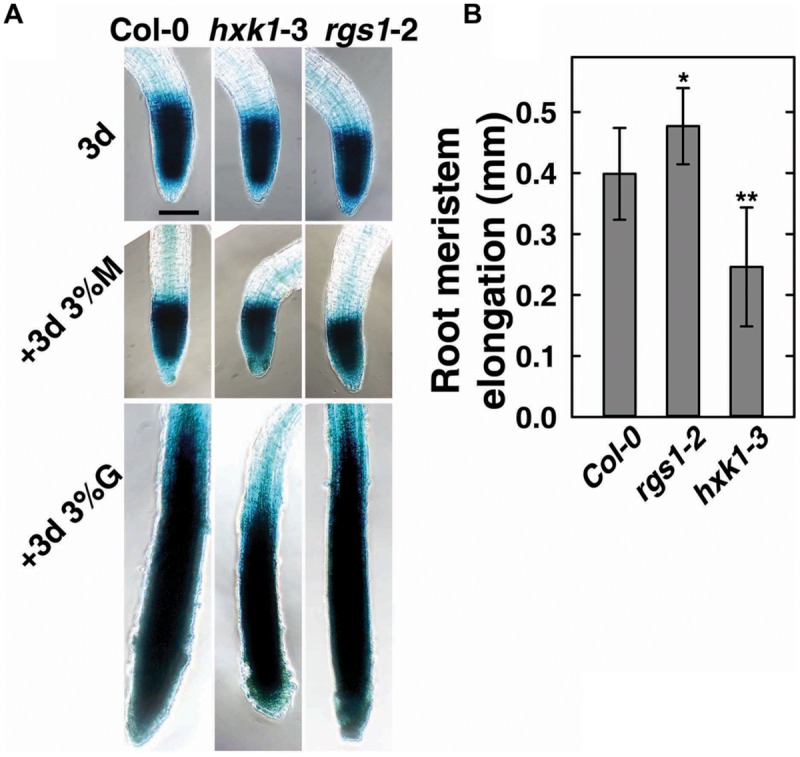
**Cell cycle progress in *rgs1*-2 or *hxk1*-3 mutant showed changed response to D-glucose. (A)** Meristematic activity in the primary root tip of Col-0, *hxk1*-3 and *rgs1*-2 plants as visualized by GUS-staining under the indicated sugar conditions as described in Section “Materials and Methods.” Seeds were germinated and cultured in 1/2 MS liquid medium for 3 days (3d); and then transferred to 1/2 MS liquid medium supplemented with 3% (w/v) D-glucose(+3d 3%G) or 3% (w/v) D-mannitol (+3d 3% M) for 3 more days. Scale bar = 0.1 mm. **(B)** The root meristem elongation as determined by the length-change of GUS-staining region in the primary root tip of Col-0, *hxk1*-3 and *rgs1*-2 seedlings. Values are means ± SD (*n* = 8–12) from one representative experiment of 3 biological replications. Pairwise Student’s *t*-test was used to compare values to the Col-0. ^∗^*P* < 0.05; ^∗∗^*P* < 0.01.

To verify the role of AtRGS1and AtHXK1 in meristematic cell activity, and to extend a previous study on the behavior of glucose sensors in sugar promoting meristem activation (Xiong et al., 2013), we compared the cell cycle activity in the primary roots of Col-0, *hxk1*-3 and *rgs1*-2 single mutant lines (**Figure 3**). The steady-state level of A-type CDK—CDKA;1, which is constitutively high in actively dividing cells was investigated using existing GUS reporter genes which were stably integrated into the wild-type (West et al., 2004; Skylar et al., 2011) or mutant backgrounds (this study). In order to minimize the influence of endogenous sugar from the endosperm or photosynthesis, seeds were germinated in sugar-free liquid medium, then 3-day-old seedlings were transferred to fresh liquid medium supplemented with 3% D-glucose or D-mannitol for another 3 days treatment as previously described (Zhang et al., 2010; Xiong et al., 2013). As shown in **Figure 3A**, D-glucose, but not D-mannitol, activated the *CDKA;1* promoter in all three genotypes. Quantitative analysis showed that the *rgs1*-2 mutant had increased (*P* < 0.05) root meristem size in response to glucose as determined by GUS-staining, whereas the *hxk1*-3 mutant had decreased (*P* < 0.01) size (**Figure 3B**), consistent with the phenotypes we observed in glucose-promoted root growth (**Figure 2A**). Moreover, it indicated an overlap in function between AtRGS1 and AtHXK1 in root meristem modulation in response to glucose.

### AtHXK1 Operates Downstream of Rapid Glucose-induced AtRGS1 Endocytosis

**FIGURE 4:**
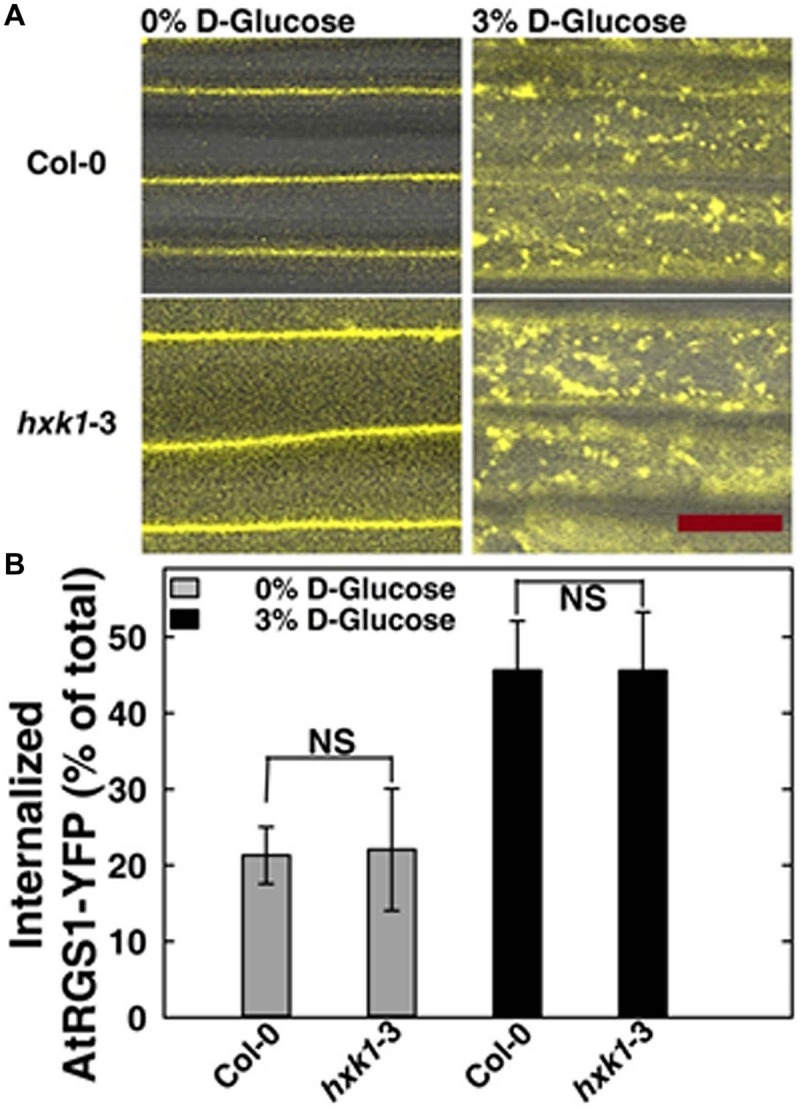
**AtRGS1 internalizes in response to D-glucose even in the absence of AtHXK1. (A)** Hypocotyls cells of 7-day-old Col-0 and *hxk1*-3 *Arabidopsis* seedlings stably expressing AtRGS1-YFP imaged after treatment with 3% D-glucose (w/v) for 30 min. Scale bar = 20 μm. **(B)** Quantization of the percentage of AtRGS1-YFP internalized in epidermal cells of Col-0 and *hxk1*-3 mutant seedlings hypocotyls before and after glucose stimulation. Values are percentage means ± SD, from a representative experiment (*n* = 10) of 3 biological replications. ANOVA single factor analysis (α = 0.05) was conducted to compare the difference between plant lines with the same glucose treatment.

In *Saccharomyces cerevisiae*, glucose activation of cAMP synthesis (cAMP/PKA pathway) requires both the presence of a G-protein-coupled receptor system for extracellular glucose sensing, and phosphorylation of the sugar by hexokinases (HXKs, Hxk2/Snf1/Mig1 pathway) for cytoplasmic glucose sensing (Rolland et al., 2000). While the exact mechanism in this process is unknown, the data indicate a functional connection between G-proteins and hexokinases. To test if an analogous relationship between extracellular and cytoplasmic glucose perception exists in *Arabidopsis*, we quantitated glucose-induced activation of G signaling. The most rapid *in vivo* reporter for G protein activation currently available is glucose-induced endocytosis of AtRGS1 which occurs in minutes (Urano et al., 2012; Fu et al., 2014). Specifically, AtGPA1 (Gα) is self-activating through spontaneous binding of GTP but is held in the resting state by the inhibitory action of its physical partner, AtRGS1. Glucose relieves this inhibition by causing physical de-coupling of AtRGS1 and AtGPA1 through WNK kinase-dependent phosphorylation at the C terminus of AtRGS1 (Urano et al., 2012). Phosphorylation of AtRGS1 drives its endocytosis thus physically uncoupling AtRGS1 from AtGPA1 and allowing activation of G protein signaling (Urano et al., 2012; Fu et al., 2014). As shown in **Figure 4**, loss of *AtHXK1* did not affect the level of glucose activated AtRGS1 re-localization, confirming that the regulatory role of AtHXK1 in AtRGS1-dependent *TBL26* induction shown in **Figure 1A** lies downstream of the apical event in G protein coupled signaling.

### RHIP1 is a Nexus in AtRGS1- and HXK1-dependent Sugar Sensing

**FIGURE 5:**
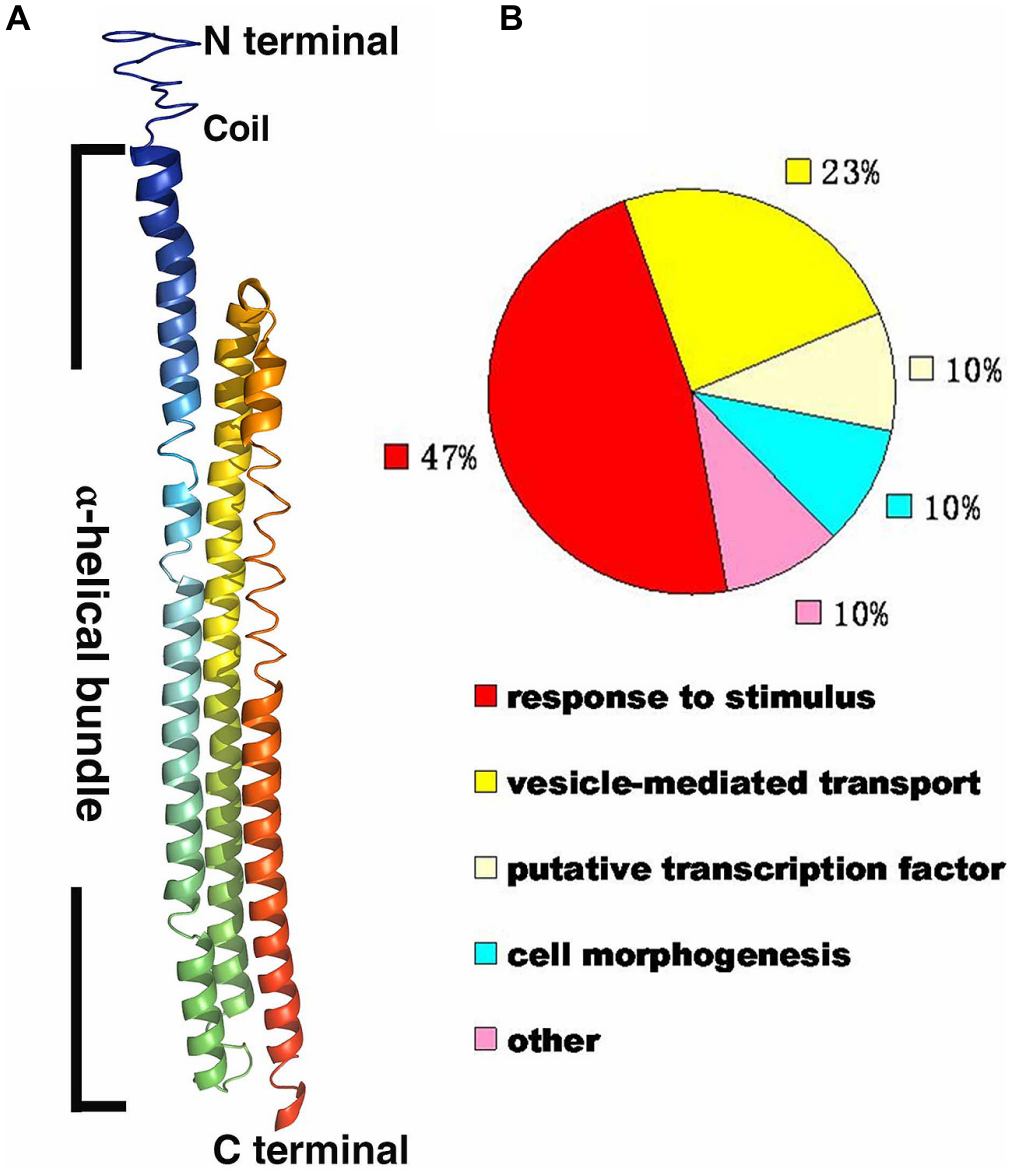
**Bioinformational analysis of RHIP1 (At4g26410). (A)** A modeled structure of *Arabidopsis thaliana* RHIP1. The structure was predicted by the I-TASSER program based on the RHIP1 protein sequence, and processed by PyMOL software. The three α-helical bundleis shown with rainbow colors, and the top is the N-terminal head composed of coils. **(B)** Functional analysis of 21 RHIP1 interacting proteins are annotated as “response to stimulus” (47%). These 21 proteins are AT2G32670, AT5G05760, AT1G35720, At2g26400, At2g42400, AT1G28520, AT1G20100, AT2G26300, AT4G17730, AT1G05500, AT3G26090, AT3G49290, At5g42030, AT3G60600, AT5G14240, AT5G43850, AT5G03540, AT3G59220, AT4g13640, AT5g67380, and AT4G29130. The function cluster analysis was performed based on TAIR GO annotations.

**FIGURE 6:**
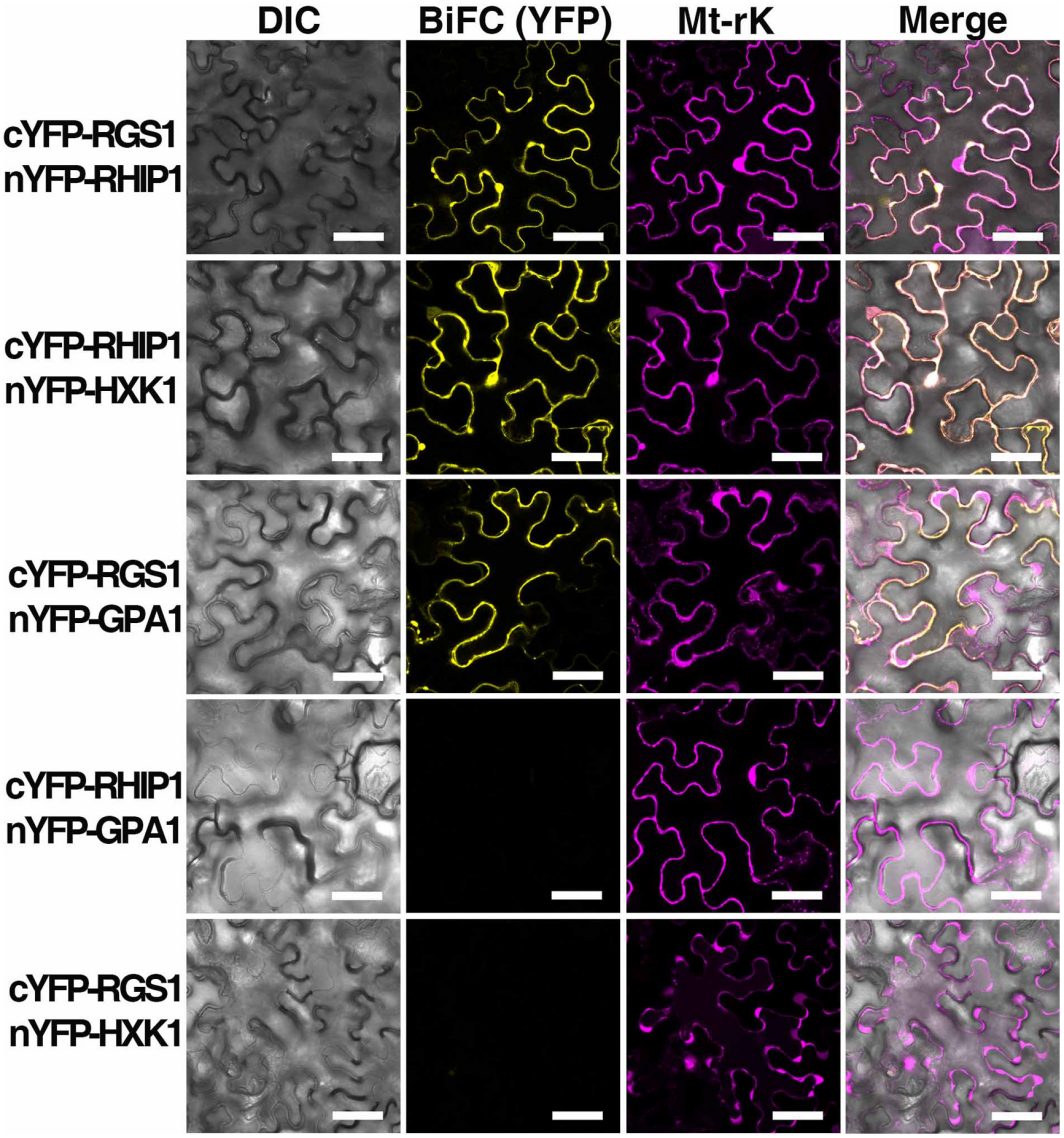
***In vivo* interaction between AtRGS1 and RHIP1, and AtHXK1 and RHIP1 determined by physical complementation of YFP using Bimolecular Fluorescence complementation (BiFC).** The test pairs are indicated on the left. **(Column 1)** Differential interference contrast (DIC) images of transformed cells. **(Column 2)** BIFC (YFP). cYFP-tagged proteins were co-transformed with nYFP-tagged proteins into tobacco leaves as described in Section “Materials and Methods.” **(Column 3**) Mt-rk is an RFP mitochondria marker used for the transformation control. Fluorescence complementation of split YFP and expression of Mt-rk were observed by confocal fluorescence microscopy. **(Column 4)** DIC, BiFC, and Mt-rk are merged. Scale bars = 50 μm.

A deep screen for *Arabidopsis* G-protein interacting proteins (Klopﬄeisch et al., 2011) yielded an uncharacterized conserved protein encoded by *At4g26410* that interacts with both AtRGS1 and AtHXK1 (Supplementary Figure S4). We investigated the possibility that this protein provides a functional connection between AtHXK1 and AtRGS1 glucose signaling. We designated this protein ***R***GS1 and ***H***XK1 ***I***nteracting ***P***rotein 1 (RHIP1). RHIP1 contains an *U*ncharacterized *C*onserved “*P*rotein” domain (UCP022280) with only one other protein (At2g45060) containing this domain in *Arabidopsis*. To our knowledge, these two proteins have not previously been identified in any study. The predicted protein structure of RHIP1 is a three-stranded helix with an N-terminal head composed of coils (**Figure 5A**). The predicted GO biological process of RHIP1 protein based on global and local protein similarity includes response to fungicide, receptor internalization, and regulation of receptor recycling (I-TASSER server) (Zhang, 2008; Roy et al., 2010, 2012). The top 1 enzyme homolog of RHIP1 protein in the Protein Database is the *Escherichia coli* histidine kinase sensor TorS sensor domain involved in signal transduction (Zhang, 2008; Moore and Hendrickson, 2009; Roy et al., 2010, 2012). RHIP1 interacts with 21 other proteins in the G protein interactome (Klopﬄeisch et al., 2011). Based on TAIR gene ontology annotations, 47% of these proteins are predicted to be involved in response to stimulus (**Figure 5B**)^3^. This finding prompted us to hypothesize that RHIP1 may have a role in sugar signaling through interactions with HXK1 and AtRGS1. To confirm interactions of RHIP1 with both AtRGS1 and AtHXK1 in plant cells, we used BiFC performed as described by Klopﬄeisch et al. (2011). As shown in **Figure 6**, AtRGS1 and AtHXK1 interacted with RHIP1 *in vivo*. Two negative and one positive control were included. The positive control was AtRGS1 and AtGPA1. The two negative controls were RHIP1-AtGPA1 and AtRGS1-AtHXK1 pairs. While *in vivo* interaction is confirmed, we do not reach a conclusion on the subcellular location of this interaction. Tobacco pavement cells, due to the large vacuole, are not suitable for subcellular location analysis. The dynamics of the confirmed interactions is not clear. While the data show that RHIP1 interacts with both AtRGS1 and AtHXK1, they do not show that this interaction is simultaneous; neither do the data preclude a scaffold role of RHIP1 to bring together AtRGS1 and AtHXK1. This is because that while AtRGS1 is located on the plasma membrane and the endomembrane system, AtHXK1 subcellular location is broad, including the cytoplasm, the mitochondrion and the nucleus and therefore it is plausible that RHIP1 is a protein complex scaffold.

### RHIP1 Participates in Glucose Signaling in *Arabidopsis*

**FIGURE 7:**
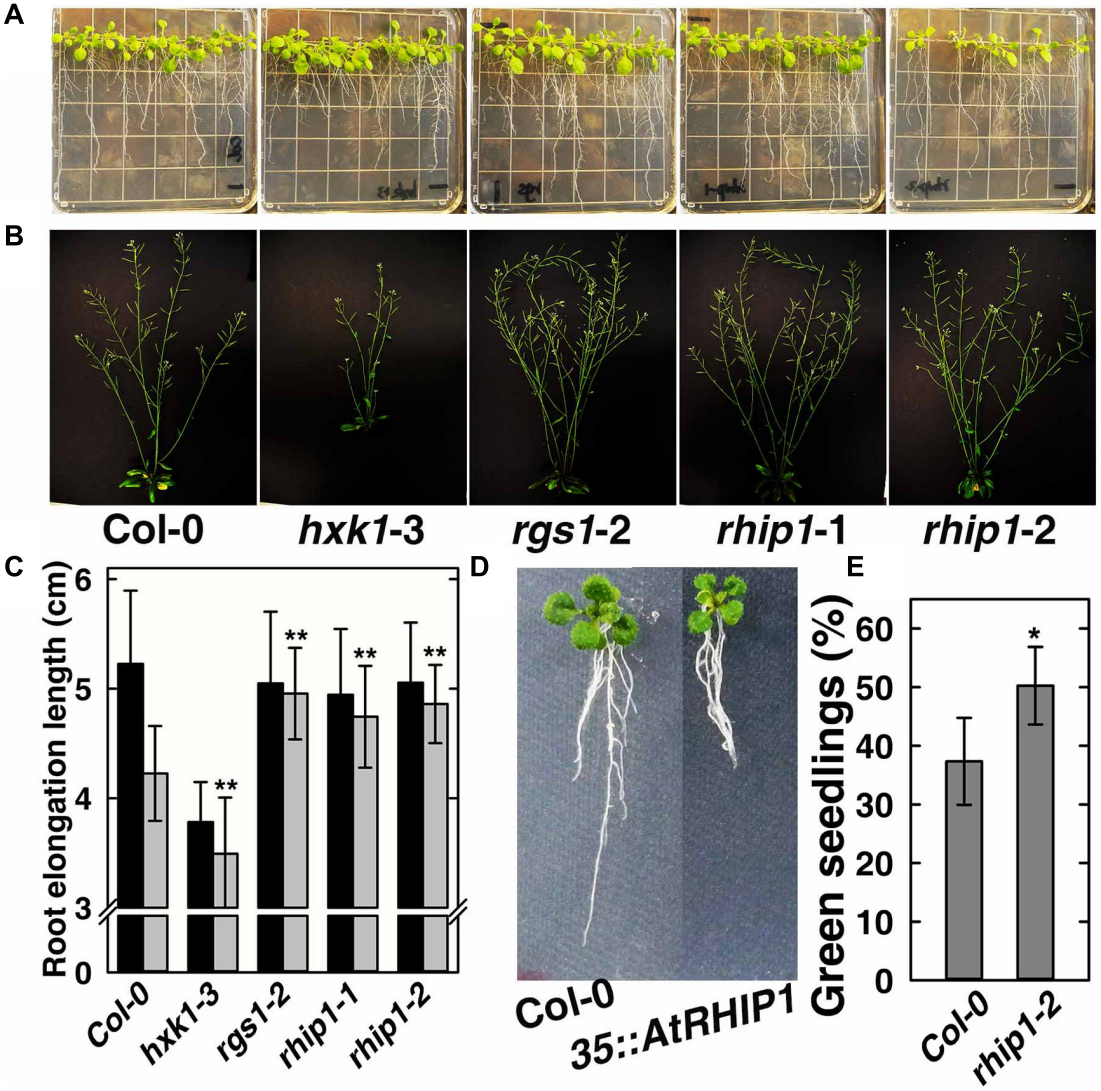
***rhip1* mutants share growth phenotypes with the *rgs1*-2 null mutant. (A)** 2-week-old Col-0, *hxk1*-3, *rgs1*-2, *rhip1*-1, and *rhip1*-2 plants grown in 1/4 × MS medium supplemented with 1% D-glucose (w/v) and grown at 200 μmol s^-1^ m^-2^ in 8/16 h (L/D). **(B)** 5-week-old Col-0, *hxk1*-3, *rgs1*-2, *rhip1*-1, and *rhip1*-2 plants grown at 160 μmol s^-1^ m^-2^ in 16/8 h (L/D). **(C)** Comparison of root elongation of Col-0, *hxk1*-3, *rgs1*-2, *rhip1*-1, and *rhip1*-2 seedlings transferred to 1/4 × MS medium supplemented with 0.25% (black) or 3% (gray) w/v D-glucose and grown at 70 μmol s^-1^ m^-2^ in 16/8 h (L/D). Values indicate means ± SD (*n* = 10–18) from a representative experiment. For the 3% D-glucose treatment, ANOVA single factor analysis (α = 0.05) was conducted to compare values to the Col-0. ^∗∗^*P* < 0.01. **(D)** 2-week-old Col-0 and *35S::YFP-RHIP1* plants grown in 1/4 × MS medium supplemented with 3% D-glucose (w/v). **(E)** Green seedling assay of the *rhip1-*2 mutant. The assay was performed as described in Section “Material and Methods.” The average percentage of seedlings showing green cotyledons was determined and presented with means ± SD from one representative experiment of 4 biological replications. ANOVA single factor analysis (α = 0.05) was conducted to compare values to the Col-0. ^∗^*P* < 0.05.

In order to elucidate the physiological role of RHIP1 in glucose signaling, two *RHIP1* T-DNA insertion mutants, *rhip1*-1 and *rhip1*-2 were generated (Supplementary Figure S5). Plants homozygous for *rhip1*-1 and *rhip1*-2 were confirmed to be transcript null. Both *rhip1*-1 and *rhip1*-2 mutants had longer roots in young seedlings and qualitatively larger inflorescence in adult plants (**Figures 7A–C**); these are phenotypes shared by the *rgs1*-2 mutant. Over-expression of *RHIP1* in Col-0 resulted in defects in root elongation (**Figure 7D**). Acute doses of glucose (6%, w/v) cause *Arabidopsis* seedling development to arrest and this phenotype is attenuated in both *HXK1* and *RGS1* loss-of-function mutants (Moore et al., 2003; Chen and Jones, 2004). We used this standardized green seedling assay to explore the role of RHIP1. As shown in **Figure 7E**, *rhip1-*2 mutants showed less sensitivity to 6% glucose than Col-0 seedlings. These *rhip1* phenotypes were not the consequences of altered transcript level for *RGS1* or *HXK1* (Supplementary Figure S2). As shown in **Figures 1A–C**, genetic ablation of *RHIP1* increased glucose-induced *TBL26* expression (around 4.5 fold), while there was no effect on the expression of *CA2* or *DIN1*.

Since it is predicted that AtHXK1 operates on AtRGS1 signaling downstream of the rapid glucose-induced AtRGS1 endocytosis (**Figures 1A** and **4**), we explored the role of their common interactor —AtRHIP1 in this process. As expected, loss of *AtRHIP1* did not affect the level of glucose activated AtRGS1 re-localization (Supplementary Figure S6), consistent with the regulatory role of AtHXK1 localized downstream of the apical event in G protein coupled signaling.

In summary, a partial functional conversation occurring between AtRGS1 and AtHXK1 was shown by glucose-regulated gene expression, epistasis analyses, and protein–protein interaction: (1) AtRGS1 and AtHXK1 showed hierarchical relationships in regulation of *TBL26, CA2*, and *CAB2* expression, suggesting collaboration between AtRGS1 and AtHXK1 in regulating gene expression. (2) *hxk1* mutant exhibited the opposite root elongation and leaf expansion phenotype to the *rgs1* mutants, while the double mutant showed an intermediate phenotype, implying a feed-back relationship between AtRGS1 and AtHXK1 in controlling cell activity. This was confirmed by the opposite behavior in glucose promotion of root meristem activation. The intermediate response in the double mutant was not additive since the phenotype of the single mutants were opposite. (3) Loss of *AtHXK1* abrogated AtRGS1-dependent signaling as measured by *TBL26* expression without changing the level of glucose activated AtRGS1 endocytosis, indicating that AtHXK1 acts downstream of AtRGS1 in sugar-activated AtRGS1 signaling. (4) AtRGS1 and AtHXK1 have a common protein interactor, RHIP1, a predicted signaling component, providing a direct nexus in AtRGS1- and AtHXK1-dependent sugar sensing. (5) Gene expression and seedling growth phenotype analyses of the *rhip1* mutant confirmed a potential role in AtRGS1 and AtHXK1 signaling.

### Accession Numbers

AtRGS1, At3g26090; TBL26, At4g01080; AtHXK1, At4g29130; AtCA2, At5g14740; AtTUB4, At5g44340; AtDIN1, At4g35770; RHIP1, At4g26410; CAB2, At1g29920. Mutant alleles for the genes were generated from the indicated T-DNA insertion lines: *hxk1-1*, SALK_034233; *hxk1-2*, CS864200; *hxk1-3*, CS861759; *rhip1-1*, SALK_091518C; *rhip1-2*, SALK_061002.

## Conflict of Interest Statement

The authors declare that the research was conducted in the absence of any commercial or financial relationships that could be construed as a potential conflict of interest.

## Footnotes

1 http://zhanglab.ccmb.med.umich.edu/I-TASSER/

2 http://www.arabidopsis.org/tools/bulk/go/index.jsp

3 http://www.arabidopsis.org/tools/bulk/go/index.jsp
