# Supplementary material for: Cooperative control between AtRGS1 and AtHXK1 in a WD40-repeat protein pathway in Arabidopsis thaliana
Source: Front Plant Sci. 2015 Oct 13;6:851. doi: 10.3389/fpls.2015.00851 (PMC4602111; doi:10.3389/fpls.2015.00851)
Supplement: Supplementary file 1 [file Data_Sheet_1.PDF]

Supplemental Materials

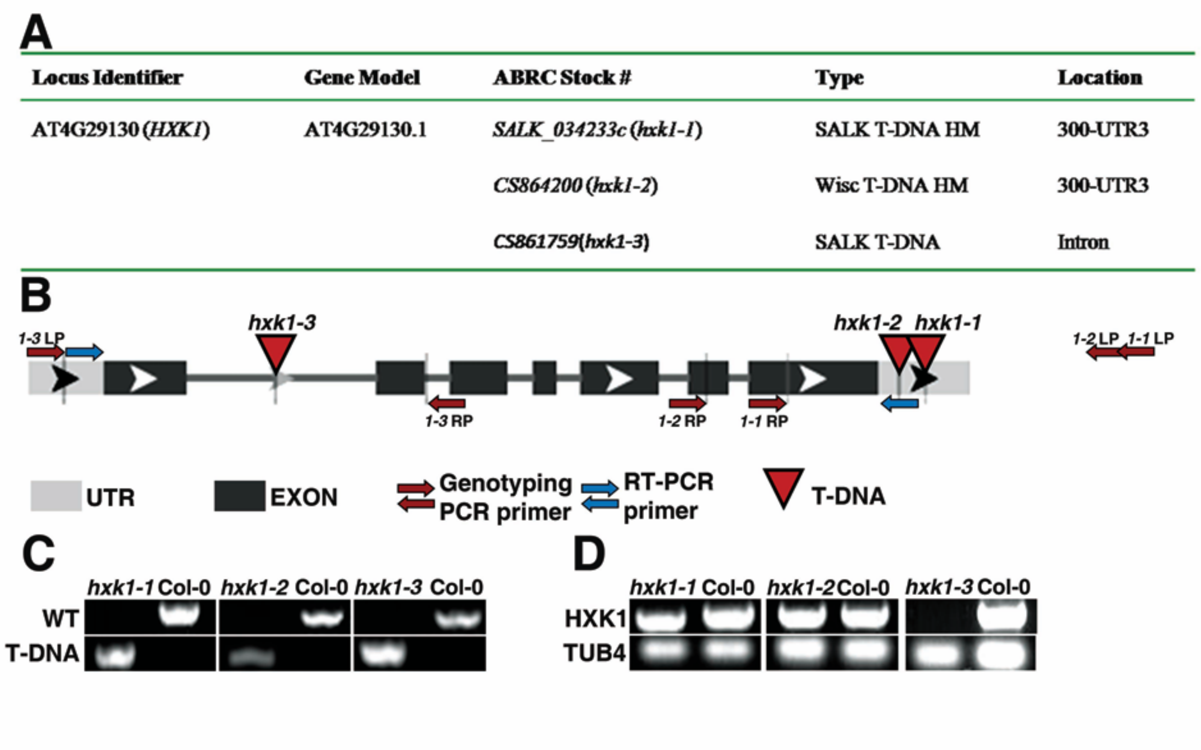

Figure S1 Molecular analysis of the *hxk1* mutant lines.

(**A**) The *T-DNA* insertion mutant lines of *AtHXK1* in Col background. (**B**) T-DNA insertion mutants of *hxk1*-1, *hxk1*-2, and *hxk1*-3. Red triangles show the T-DNA insertion site, and it respects *hxk1*-3, *hxk1*-2, and *hxk1*-1 from left to right; gray boxes represent exons; red arrows indicate the position of primers used for genotyping; blue arrows indicate the position of primers used for RT-PCR. (**C**) Genotyping results using a primer from the left border of the T-DNA to determine insertion of the T-DNA (T-DNA) or without the T-DNA border to amplify the wild type allele (WT). All mutants shown here are homozygous at the indicated locus. (**D**) Transcription level of *HXK1* in *hxk1*-1, *hxk1*-2, and *hxk1*-3 mutant lines. The mRNA and cDNA were prepared with RNAeasy (Qiagen) and Superscript III (Invitrogen) according to the manufacturer’s instructions.

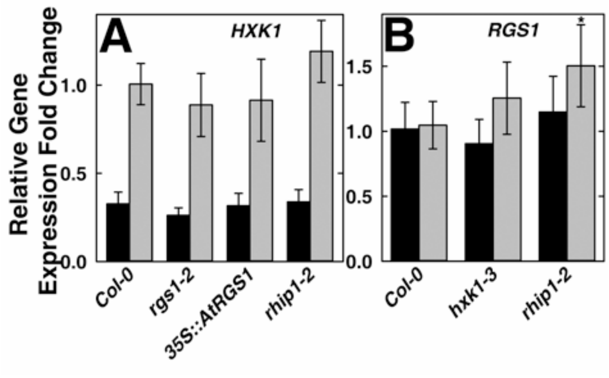

Figure S2 Quantitative Real-time PCR analysis of *HXK1* and *RGS1* in Arabidopsis seedlings.

7-day-old seedlings with the indicated genotypes were starved for 2 days then treated with 1/2×MS (0% D- glucose, shown by black bars) or 3% D-glucose (gray bars) for 3h as described in the Methods. Transcripts of *HXK1* (**A**), and *RGS1* (**B**) were quantitated using qRT-PCR. Values are the means of the fold changes ± SD of three independent biological replicates. Each biological replication had at least 3 technical replications. ANOVA single factor analysis (α=0.05) was conducted to compare the relative fold-change in gene expression of different plant lines with wild type control plants. *, significant at $P< 0.05$; **, significant at $P<0.01$.

Supplemental Materials

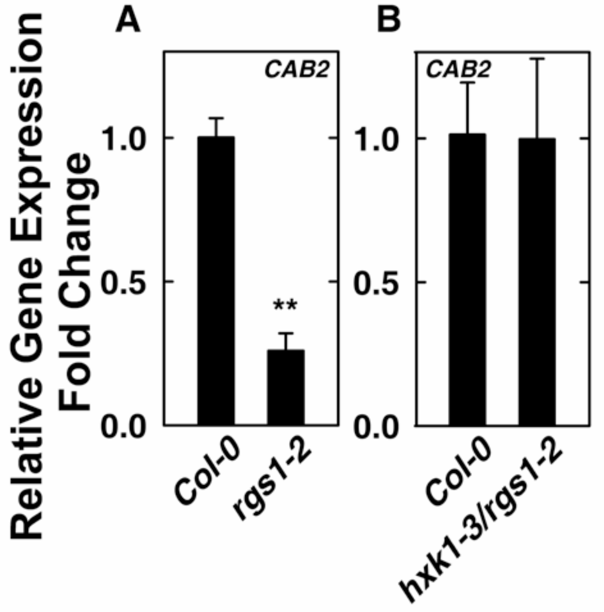

Figure S3 Quantitative Real-time PCR analysis of *CAB2* in Arabidopsis seedlings.

7-day-old seedlings with the indicated genotypes were starved for 2 days then treated with 1/2×MS supplemented with 0% D-glucose (black bars) for 3h as described in the Methods. Transcripts of *CAB2* in Col-0 and *rgs1*-2 mutant (**A**), Col-0 and *hxk1*-3/*rgs1*-2 double mutant (**B**) were quantitated using qRT-PCR. Values are the means of the fold changes ± SD of 2 or 3 biological replications. Each biological replication had at least 3 technical replications. ANOVA single factor analysis (α=0.05) was conducted to compare the relative fold-change in gene expression of different plant lines with wild type control plants. *, significant at $P < 0.05$; **, significant at $P<0.01$.

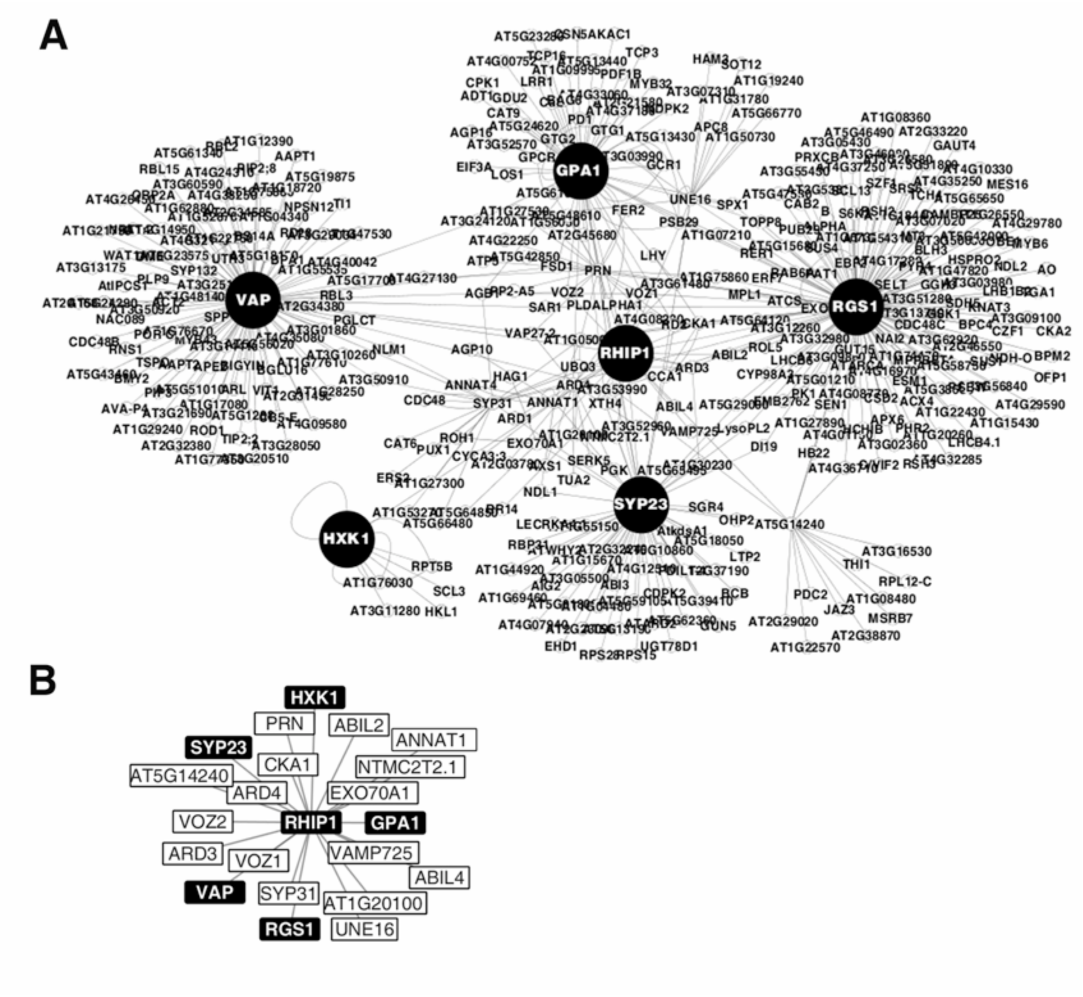

Figure S4 AtRGS1 and AtHXK1 Interacting Protein, RHIP1 (At4g26410).

(**A**) At least 381 proteins form the RHIP1 protein interaction network in Arabidopsis, and these proteins form four main hubs: AtRGS1 (At3g26090), AtGPA1 (At2g26300), AtVAP (At3g60600), and AtSYP23 (At4g17730), respectively. (**B**) There are a total of 21 proteins, VAMP725 (At2g32670), SYP31 (At5g05760), ANNAT1 (At1g35720), ARD3 (At2g26400), VOZ2 (At2g42400), VOZ1 (At1g28520), At1g20100, GPA1 (At2g26300), SYP23 (At4g17730), NTMC2T2.1 (At1g05500), RGS1 (At3g26090), ABIL2 (At3g49290), ABIL4 (At5g42030), VAP (At3g60600), At5g14240, ARD4 (At5g43850), EXO70A1 (At5g03540), PRN (At3g59220), UNE16 (At4g13640), CKA1 (At5g67380) and HXK1 (At4g29130) directly interacting with RHIP1(At4g26410).

Supplemental Materials

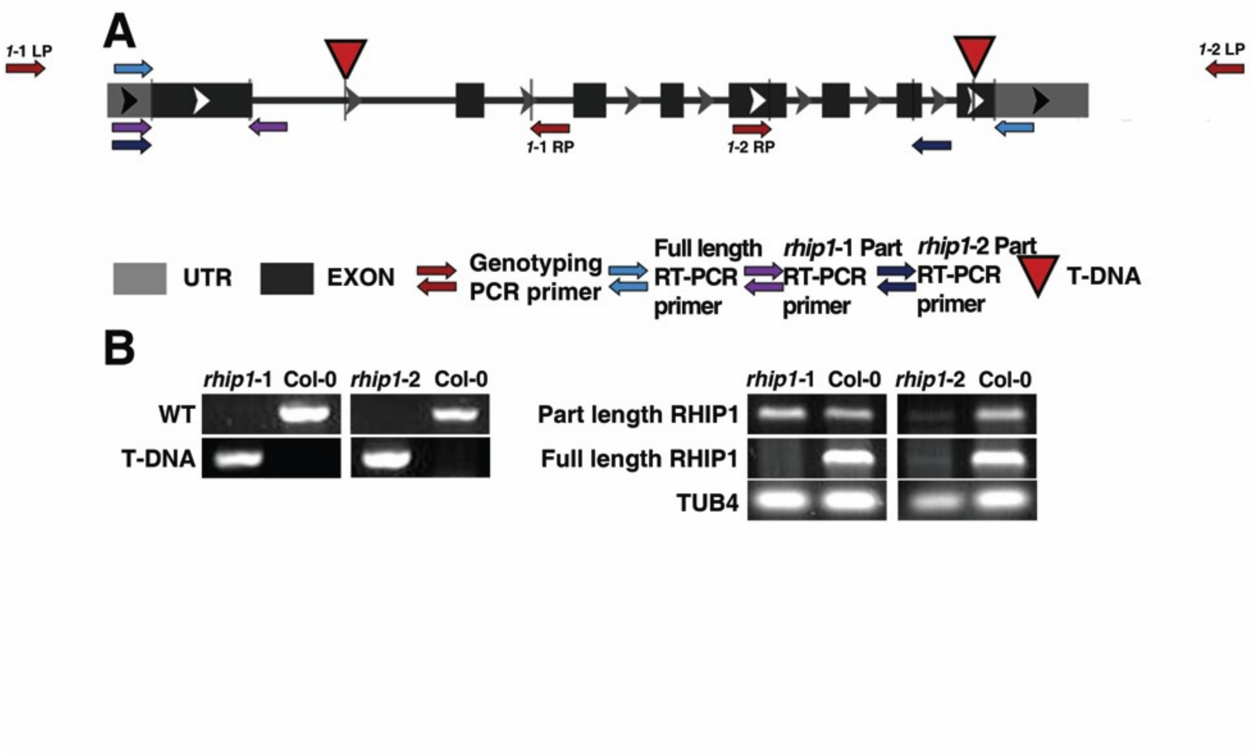

Figure S5 Molecular analysis of the *rhip1* mutant lines.

(**A**) T-DNA insertion mutants of *RHIP1*. Red triangles show the T-DNA insertion sites for mutants *SALK_091518* (*rhip1*-1) and *SALK_061002* (*rhip1*-2) from left to right; Gray boxes represent exons; Red arrows indicate the position of primers used for genotyping; Blue, purple and dark blue arrow pairs indicate the position of primers used for RT-PCR. (**B**) Genotyping results using a primer from the left border of the T-DNA to determine insertion of the T-DNA (T-DNA) or without the T-DNA border to amplify the wild type allele (WT). All mutants shown are homozygous at the indicated locus. The transcription level of full length and partial length *RHIP1* in *SALK_091518* (*rhip1*-1) and *SALK_061002* (*rhip1*-2) mutant lines is shown. The mRNA and cDNA were prepared with RNAeasy (Qiagen) and Superscript III (Invitrogen) according to the manufacturer's instructions.

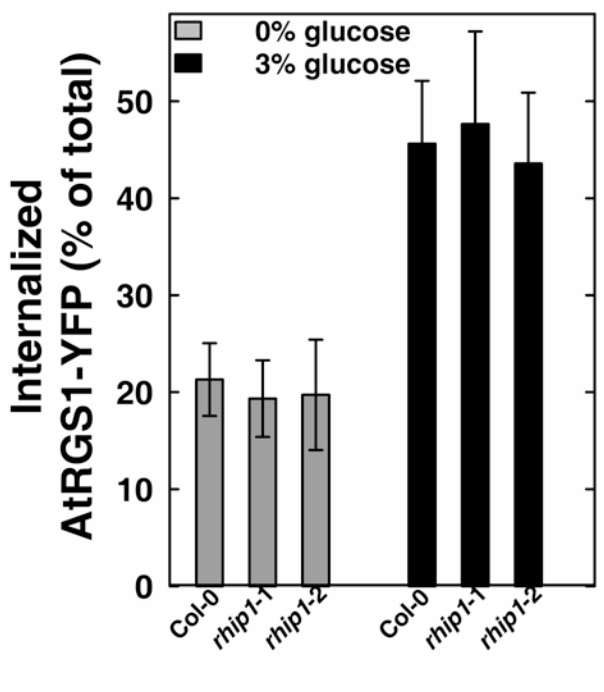

Figure S6 AtRGS1 internalizes in response to D-glucose even in the absence of AtRHIP1.

Quantization of the percentage of AtRGS1-YFP internalized in epidermal cells of Col-0 and *rhip1*-1, *rhip1*-2 mutant seedlings hypocotyls before and after glucose stimulation. Values are percentage means ± SD, from a representative experiment (n=10). ANOVA single factor analysis (α=0.05) was conducted to compare the difference between plant lines with the same glucose treatment.

Supplementary Table S1 Primers used for PCR.

Name	AGI Number	Primers (5′-3′)
hxk1-1	At4g29130	L: ATTTATGCTCATAAAGCTGGACTTG
		R: TGAGAAAAGTTGTGATCAGTCTCTG
hxk1-2	At4g29130	L: TGGTTTTTATGTGAATCATTTTGTG
		R: GTTCTTCTAAAGATGGCTGAAGATG
hxk1-3	At4g29130	L: TTGATTATTTCTTCTTTCTGGCTTG
		R: AGAACAGAAAACTGACATCTGAACC
LBb1.3		ATTTTGCCGATTTCGGAAC
pWiscDsLox LB		AACGTCCGCAATGTGTTATTAAGTTGTC
Full AtHXK1	At4g29130	F: ATGGGTAAAGTAGCTGTTGG
		R:TTAAGAGTCTTCAAGGTAGAGAGAGTG
rhip1-1	At4g26410	L: CTTCCTCCTTCTTTTAGATCTCCAC
		R: ATAAAAGTTTTGTGGCAGCTATCTG
rhip1-2	At4g26410	L: GATCAACGGACATCAAAGAGATC
		R: CTTTAGCAGAGAAGGATATGAAACG
Full AtRHIP1	At4g26410	F: ATGAGCGAAACCGAAGCAA
		R:TCAGACGGAAACTCCTAGGTCCGACAT
Part AtRHIP1 for rhip1-1	At4g26410	F: ATGAGCGAAACCGAAGCAAC
		R: AGCGGAACTGAGAGGTAGAA
Part AtRHIP1 for rhip1-2	At4g26410	F: ATGAGCGAAACCGAAGCAAC
		R: GCATCCCTTCCAGGAATTTG
TUB4	At5g44340	F: AGAGGTTGACGAGCAGATGA
		R: ACCAATGAAAGTAGACGCCA
TBL26	At4g01080	F: CGCCATCGAACCTTCGTCAAATTC
		R: TCGTCCATTCAATAGGCAGTTCTGA
HXK1	At4g29130	F: TGCTGCTTTCTTTGGCGATACAGT
		R: TGCTCCCAACAATCTTCAAGTCTGG
RGS1	At3g26090	F: CAATAGAAATGGCGAGTGGATGTGC
		R: CGAGGAGCCTTATGAATCAAACACG
CA2	At5g14740	F: GATGCCTTCGTGGTTCGTAATATCG
		R: TGCCCTATCACCACAATGTTTTCC
CAB2	At1g29920	F: CCAGGAACGGAGTCAAGTTTGGA
		R: CCAAAATGCTCTGAGCGTGAACC
DIN1	At4g35770	F: CAGAGTCGGATCAGGAATGG
		R: ATTTGACCGCTCTCACAACC
